# Cross-talk between lactate metabolism and immunity reveals CEP55 as a potential regulator in the immunosuppressive microenvironment of hepatocellular carcinoma

**DOI:** 10.1016/j.gendis.2024.101399

**Published:** 2024-08-28

**Authors:** Jie Li, Yuyuan Zhang, Jinhai Deng, Siyuan Weng, Hui Xu, Yuhao Ba, Anning Zuo, Shutong Liu, Quan Cheng, Jian Zhang, Peng Luo, Zhen Li, Xinwei Han, Zaoqu Liu

**Affiliations:** a Department of Interventional Radiology, The First Affiliated Hospital of Zhengzhou University, Zhengzhou, Henan 450052, China; b Interventional Institute of Zhengzhou University, Zhengzhou, Henan 450052, China; c Interventional Treatment and Clinical Research Center of Henan Province, Zhengzhou, Henan 450052, China; d Richard Dimbleby Laboratory of Cancer Research, School of Cancer & Pharmaceutical Sciences, King's College London, London WC2R 2LS, UK; e Department of Neurosurgery, Xiangya Hospital, Central South University, Changsha, Hunan 410008, China; f Department of Oncology, Zhujiang Hospital, Southern Medical University, Guangzhou, Guangdong 519015, China; g Institute of Basic Medical Sciences, Chinese Academy of Medical Sciences and Peking Union Medical College, Beijing 100730, China

The interaction of lactate metabolism with immunity plays a crucial role in the remodeling of the immune microenvironment and even in the heterogeneous progression of hepatocellular carcinoma (HCC). The intratumor-accumulated lactate served a vital role in the inefficacy of antitumor immune responses, the aggressiveness of tumor cells, and immunotherapy.^1^ Furthermore, lactate generated from the tumor microenvironment can be used as fuel for the proliferation and infiltration of immunosuppressive cells.^2^ Previous studies regarding the taxonomies of HCC, solely from the perspective of lactate^3^ or tumor immune microenvironment^4^ may introduce the potential for bias in the comprehension of HCC heterogeneity. Thus, deciphering the crosstalk properties between lactate and immune is imperative.

In this study, we aimed to explore the crosstalk between lactate metabolism and immunity in HCC. We identified and validated lactate-immune-based subtypes (LIBS) in 1378 patients from six cohorts (Table S1). Our workflow is illustrated in Figure S1. Immune cell abundance and lactate metabolic dysregulation were widely observed in HCC samples compared with normal samples (Fig. 1A, B). To further explore the potential interactions between immunity and lactate metabolism, the correlation analyses between lactate-related pathways and immune cells were implemented. This exhibited that activation of the lactate pathways tended to be positively correlated with immunosuppressive cells, implying that elevated lactate levels might contribute to the establishment of tumor immunosuppressive microenvironment (Fig. 1C; Fig. S2A).

**Figure 1:**
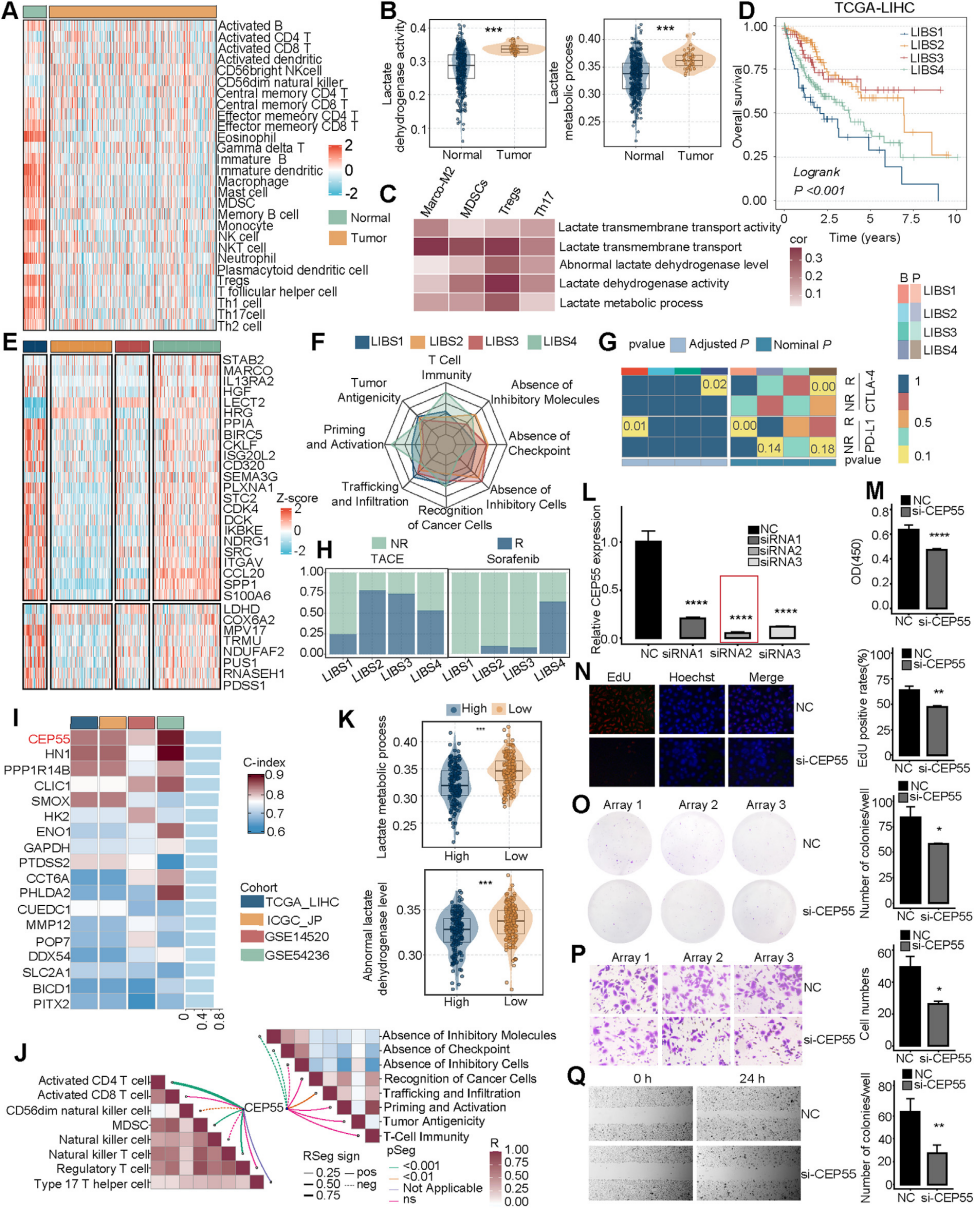
Development of hepatocellular carcinoma (HCC) lactate-immune-based subtypes (LIBS) and identification and validation of prognostic key biomarker. **(A)** The distribution difference of the infiltration of 28 immune cells between normal and tumor groups. **(B)** The distribution of lactate-related pathways between normal and tumor groups. **(C)** The correlation between lactate-related pathways and immunosuppressive cells. **(D)** Kaplan–Meier curves of overall survival among LIBS. **(E)** Heatmap of gene expression patterns of prognostic-associated lactate-related genes (LRGs) and immune-related genes (IRGs) in distinct clusters. **(F)** The radar plot of the differences in the anti-cancer immune status and immunogenicity among LIBS. **(G)** SubMap analysis revealed the potential of immune checkpoint inhibitor (ICI) therapy. **(H)** Transcatheter arterial chemoembolization (TACE) and sorafenib response ratio of LIBS. **(I)** Selection of the feature gene for LIBS1 according to the highest mean receiver operating characteristic (ROC) statistic. **(J)** The correlation between *CEP55* and immune cells/immune-related signatures. **(K)** The distribution of lactate-related pathways between high and low *CEP55* expression levels. **(L)** The interference effects on *CEP55* expression after silencing of *CEP55* with different small interfering RNAs (siRNAs) in HCC cell lines with relative *CEP55* expression determined by quantitative real-time PCR (qRT-PCR) analysis. **(M**–**Q)** Cell counting kit-8 (CCK-8) proliferation assay (M), 5-Ethynyl-2′-deoxyuridine (EdU) incorporation assay (N), colony formation assay (O), transwell migration assay (P), scratch assay (Q) of HCC cell lines. ∗*P* < 0.05, ∗∗*P* < 0.01, ∗∗∗*P* < 0.001.

Subsequently, we retrieved immune- and lactate-related genes (Table S2) and identified prognostic-associated lactate-related genes (LRGs) and immune-related genes (IRGs) using univariate Cox analysis (Supplementary Methods; Fig. S2B, C). The four clusters with distinct expression patterns were identified via the iClusterBayes algorithm (Fig. S2D, E). We found that most LRGs and immunosuppressive genes, especially SPP1, were highly expressed in the LIBS1. Notably, the expression of individual LRGs and IRGs showed obvious correlations (Fig. S2F). Furthermore, the robustness and reproducibility of LIBS were assessed in validation datasets through the nearest template prediction (NTP) and subclass mapping analysis (SubMap) algorithms (Fig. S2G). LIBS presented significant differences in clinical prognosis, with LIBS1 exhibiting the poorest outcome (Fig. 1D; Fig. S2H, I). Furthermore, LIBS1 and LIBS4 possessed more aggressive clinical features comprising advanced TNM stage and vascular invasion (Fig. S3A). Cox regression revealed that LIBS1 and LIBS4 were independent prognostic indicators (Fig. S3B). We also explored the correlation between LIBS and previous HCC classifications, in which LIBS1 was associated with iCluster 1 and Serum All (dismal prognosis) (Fig. S3C). Overall, LIBS1 was characterized by elevated lactate and immunosuppression with poor prognosis.

To unravel the distinctive biological processes of LIBS, we conducted enrichment analyses. LBS1 was remarkably enriched in hypoxia, glycolysis, and proliferation pathways. LIBS2 and LIBS3 were characterized by an elevated activity in fatty acid and bile acid metabolism. Notably, LIBS2 showed significant enrichment in the PPAR signaling pathway, in contrast, LIBS3 was associated with the Wnt/β-catenin pathway, indicating distinct molecular mechanisms underlying these subtypes. LIBS4 demonstrated notable enrichment in immune-related pathways and simultaneously presents distinctive angiogenesis activity (Fig. S4). Additionally, LIBS displayed distinct immune phenotypes, in which LIBS1 and LIBS4 displayed a superior abundance of immune cell infiltration with a higher proportion of immunosuppressive cells, particularly, regulatory T cells, and myeloid-derived suppressor cells. While LIBS2 and LIBS3 showed a higher proportion of immune-activated cells, encompassing M1 macrophages and natural killer cells (Fig. S5A, C). To gain an insight into the difference in tumor clearance effect among LIBS, we further investigated the immunogenicity by mapping immunogram and thus characterized the ability to clear tumor cells.^5^ LIBS2 and LIBS3 possessed the absence of inhibitory immunocytes and immune checkpoint expression. Whereas, LIBS1 and LIBS4 displayed higher T-cell immunity, recognition of cancer cells, and priming and activation of anti-tumor immune (Fig. 1F). Notably, most of the immune checkpoint molecules markedly up-regulated in both LIBS1 and LIBS4, accompanied by higher scores in the antigen presentation score and tumor inflammation signature, indicating the potential to benefit more from immune checkpoint inhibitor therapy (Fig. S5B, D). SubMap analysis demonstrated that LIBS1 and LIBS4 were more appropriate for immune checkpoint inhibitor treatment (Fig. 1G).

Prior analysis showed that LIBS1 was associated with hypoxia. However, hypoxia is a culprit of immunotherapy failure. To gain a deeper understanding of the mechanism by which LIBS1 benefits from immunotherapy, we calculated hypoxia scores for the IMvigor210 cohort utilizing hypoxia-related genes through the gene set variation analysis (GSVA) algorithm. As expected, patients unresponsive to anti-PD-L1 therapy exhibited higher hypoxia scores. Further, patients in LIBS1 were stratified into high and low hypoxia score groups using the median value (Table S3), with SubMap analysis subsequently validating the resistance of the hypoxia phenotype to anti-PD-L1 therapy (Fig. S5E, F). Additionally, we assessed the sensitivity of LIBS to transcatheter arterial chemoembolization and sorafenib treatment. LIBS2 and LIBS3 revealed sensitivity to transcatheter arterial chemoembolization, while LIBS4 benefited from sorafenib therapy coinciding with higher angiogenesis activity (Fig. 1H).

We further investigated the genomics landscape of LIBS, encompassing mutations and chromosomal variations. We noticed distinct mutational differences, with *TP53* dominating in LIBS1 and *CTNNB1* being more frequent in LIBS3. Moreover, chromosomal amplifications and deletions exhibited significant distributional differences among LIBS (Fig. S6A). The characterization of copy number variations on chromosomes, assessed in terms of bases, segments, and chromosomal arms, revealed that LIBS1 exhibited the highest burden of arm gain and focal loss, portending a pronounced genomic and chromosomal instability, echoing dismal prognosis (Fig. S6B–G).

To elucidate the prognostic mechanism of LIBS1, we screened LIBS1 characteristic genes with hazard ratio >1 and *P* < 0.05 (Fig. S5G) and further assessed the values for the area under the curve of the risk genes in the validation cohort. Ultimately *CEP55* with the highest mean receiver operating characteristic statistic was considered to be the LIBS1 prognostic key gene (Fig. 1I; Fig. S5H). Notably, *CEP55* was positively associated with an immunosuppressive phenotype with more immunosuppressive cells and inhibitory molecules and checkpoints. Moreover, a higher expression level of *CEPP55* was associated with lactate generation-related pathways, indicating a pivotal role for *CEPP55* as a hyperlactate and immunosuppressive tumor microenvironment (Fig. 1J, K). To deeply investigate the potential biological function of *CEP55* in HCC cell lines, we silenced the function of *CEP5*5 by siRNAs and found that the growth potential of HCC cells was diminished (Fig. 1L). Therefore, we performed several assays including CCK-8 assay, EdU incorporation assay, transwell migration assay, and scratch assay to verify the effect of *CEP55* on the proliferation and migration ability of HCC cells (Fig. 1M–Q). Altogether, *CEP55* knockdown significantly reduced HCC cell proliferation and migration compared with the normal control group suggesting that *CEP5*5 might serve as a prognostic potential biomarker for LIBS1.

Overall, deciphering the complex interaction of lactate metabolism with immunity in HCC provides novel insight for understanding the mechanism of microenvironment remodeling, which assists integrated clinical management and optimizes precision therapy for HCC.

## Funding

This study was supported by the 10.13039/501100017599Major Science and Technology projects of Henan Province, China (No. 221100310100).

## Author contributions

Z.Q.L., Z.L., and X.W.H. provided direction and guidance throughout the preparation of this manuscript. J.L., Y.Y.Z., and Z.Q.L. wrote and edited the manuscript. J.Z. and Q.C. reviewed and made significant revisions to the manuscript. S.Y.W., A.N.Z., Y.H.B., S.T.L., J.H.D., P.L., and H.X. collected and prepared the related papers. All authors reviewed and approved the final manuscript.

## Data availability

The datasets presented in this study can be found in online repositories. The names of the repository/repositories and accession number(s) can be found in the article.

## Conflict of interests

The authors declared no competing interests.

## Figures and Tables

**Figure S1:**
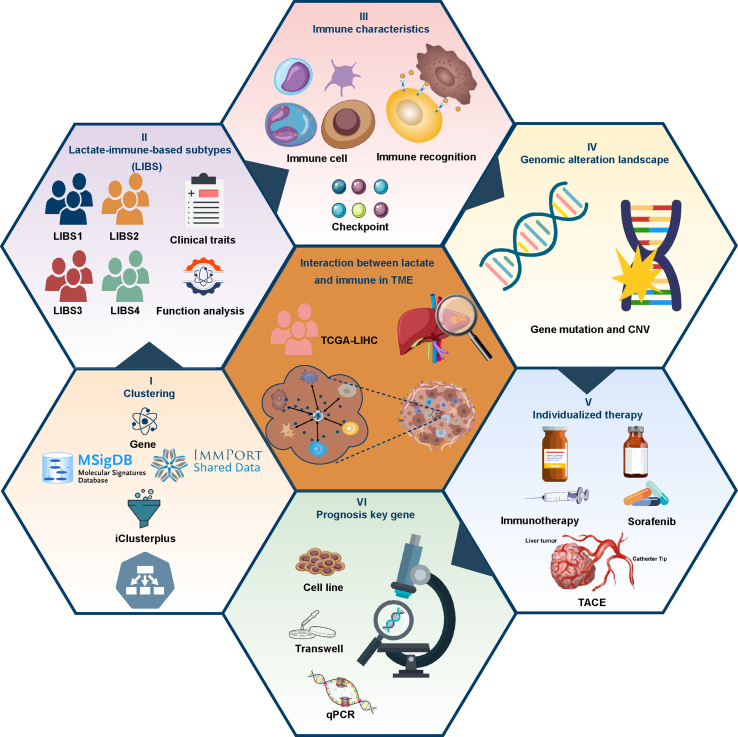
The flowchart of this study.

**Figure S2:**
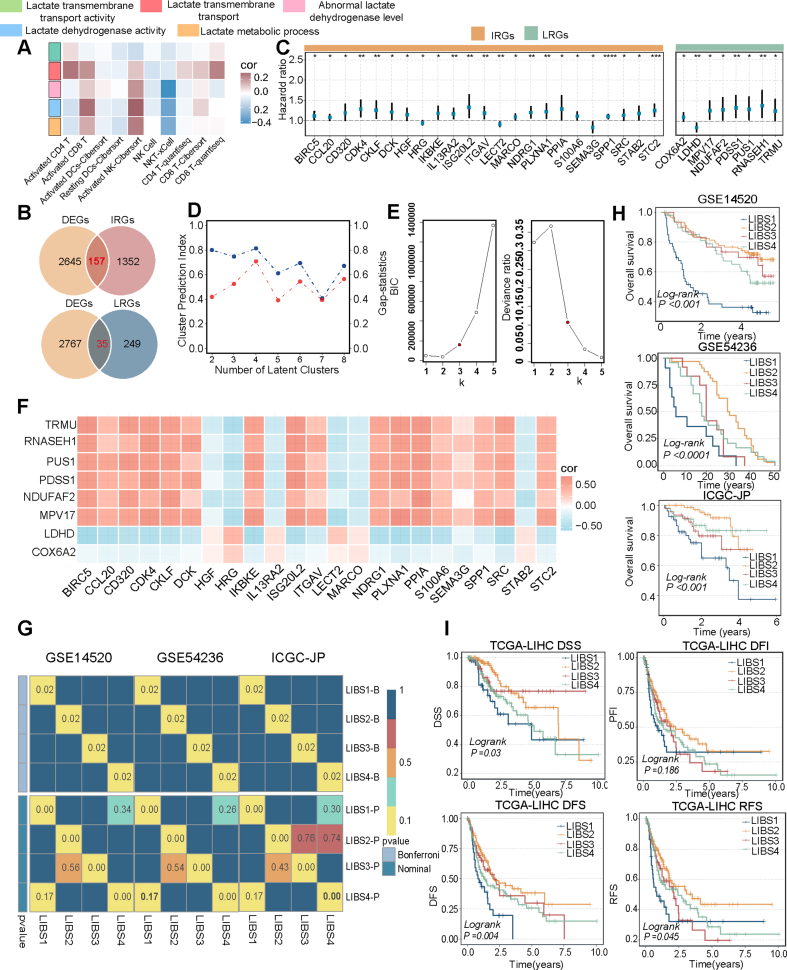
Development and validation of hepatocellular carcinoma (HCC) lactate-immune-based subtypes (LIBS). **(A)** The correlation between lactate-related pathways and immune-activated cells. **(B)** The Venn diagram exhibits the overlapping genes of differentially expressed genes (DEGs) with lactate-related genes (LRGs) and immune-related genes (IRGs), respectively. **(C)** Forest plots of LRGs and IRGs associated with prognosis based on univariate Cox regression analysis. **(D, E)** The optimal number of clusters was determined by clustering prediction index (D), Bayesian information criteria, and deviance ratio plots (*E*) between 1 and 5 clusters. **(F)** Correlation heatmaps of prognostic-associated LRGs and IRGs. **(G)** SubMap analysis reveals the similarity of gene expression profiles between the LIBS of The Cancer Genome Atlas (TCGA) cohort and validation datasets. **(H)** Kaplan-Meyer survival curves for LIBS in the validation datasets (GSE14520, GSE54236, and International Cancer Genome Consortium (ICGC-Japan)). **(I)** Kaplan-Meyer survival curves for recurrence-free survival (RFS), disease-free survival (DFS), progression-free survival (PFI), and disease-specific survival (DSS) in the TCGA cohort.

**Figure S3:**
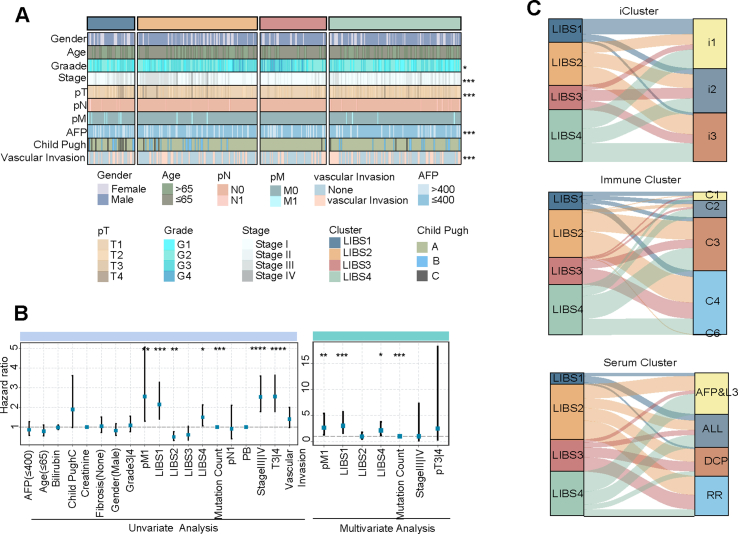
The distribution of clinical traits among lactate-immune-based subtypes (LIBS). **(A**–**C)** Correlation of LIBS with clinical characteristics and previous classifications. (A) Correlations of LIBS with clinical characteristics. (B) Forest plots for the univariant and multivariate Cox regression analysis of the clinical characteristics and the LIBS for overall survival. (C) The Sankey diagram shows the correlations between LIBS and previous classifications.

**Figure S4:**
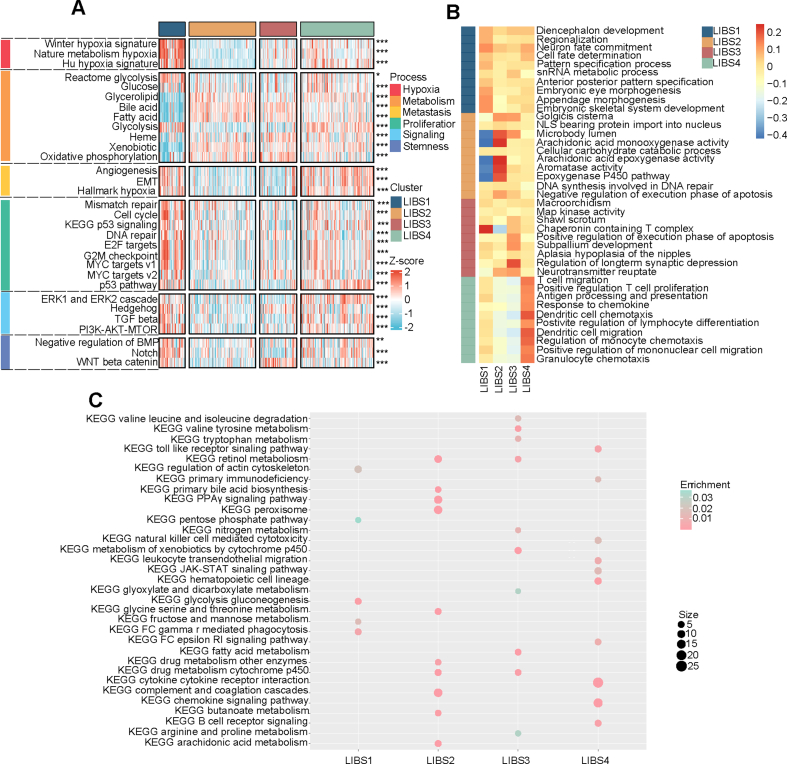
Underlying biological processes of lactate-immune-based subtypes (LIBS). **(A)** Heatmap of subtype-specific biological processes and signatures for LIBS. **(B)** Heatmap of subtype-specific Gene Ontology (GO) terms via Gene set enrichment analysis (GSEA) analysis. **(C)** Kyoto Encyclopedia of Genes and Genomes (KEGG) pathways enrichment analysis of LIBS.

**Figure S5:**
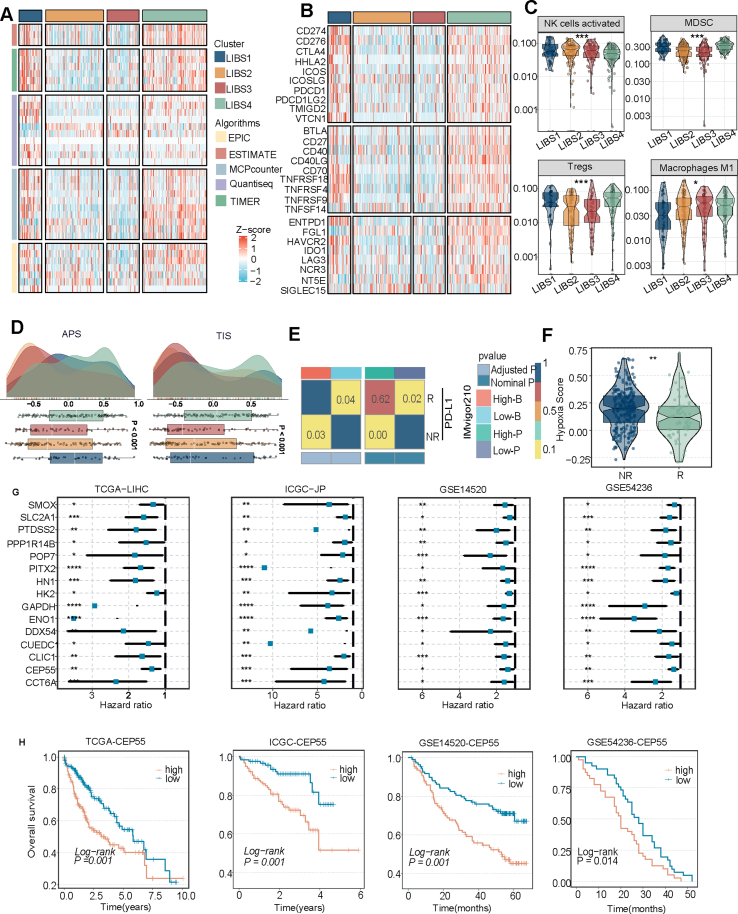
Immune landscapes and specialized therapy prediction for lactate-immune-based subtypes (LIBS) and the selection and validation of LIBS1 prognosis-related signature genes. **(A)** Heatmap of immune cell infiltration for LIBS by multiple algorithms. **(B)** Heatmap of 27 immune checkpoint molecules in all samples. Each cell represents a z-score of relative infiltration abundance or expression level. **(C)** Distribution of the immune cells (M1 macrophages and natural killer (NK) cells, regulatory T cells (Tregs), and myeloid-derived suppressor cells (MDSCs)) of LIBS. **(D)** Distributions of antigen presentation score (APS) and tumor inflammation signature (TIS) of LIBS. **(E)** SubMap analysis exhibited that the high hypoxia score group was insensitive to immune checkpoint inhibitor (ICI) therapy (Bonferroni *P* = 0.03). **(F)** Distributions of hypoxia score between no response to ICI therapy and response to ICI therapy groups. **(G)** Identification of 15 risk genes with hazard ratio (HR) > 1 and *P* < 0.05 in univariate analysis in enrolled cohorts. **(H)** Survival analysis of high and low *CEP55* expression groups in enrolled cohorts.

**Figure S6:**
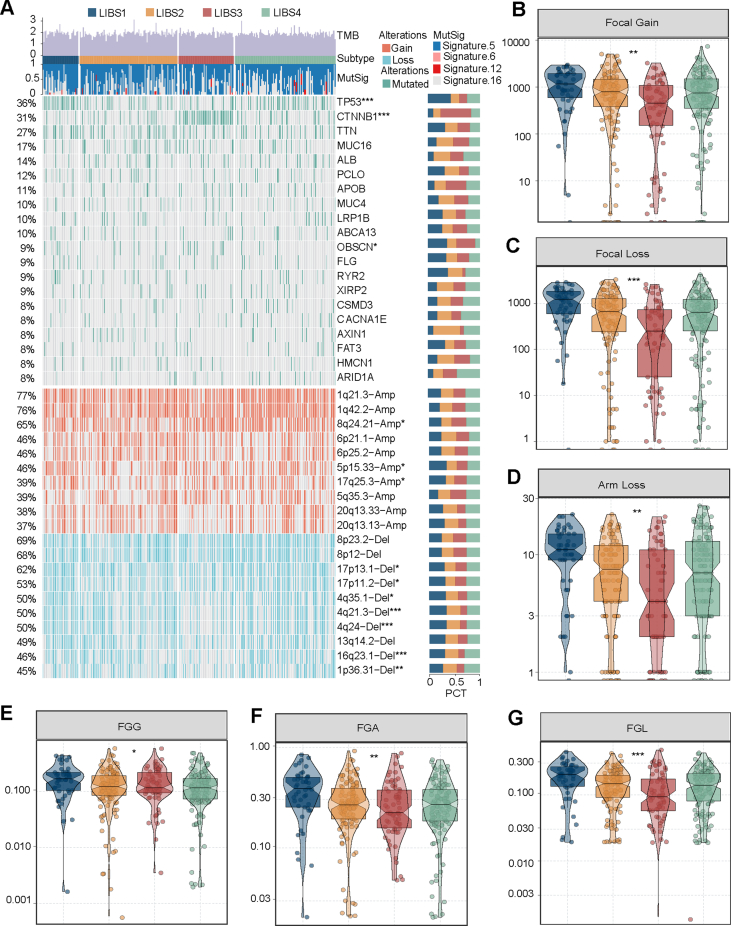
Genomic alterations of the lactate-immune-based subtypes (LIBS). **(A)** The somatic mutation landscape of the top 20 frequently mutated genes and copy number variation (CNV) landscape of the top 20 high-frequent amplification (AMP) and homozygous deletion (HOMDEL) chromosome regions for LIBS. **(B**–**G)** The distribution of focal gain (B), focal loss (C), arm loss (D), fraction of genome lost (FGL) (E), fraction of genome gained (FGG) (F), and fraction of genome altered (FGA) (G) in LIBS. ∗*P* < 0.05, ∗∗*P* < 0.01, ∗∗∗*P* < 0.001, ∗∗∗∗*P* < 0.0001.
