# Supplementary material for: Cross-talk between lactate metabolism and immunity reveals CEP55 as a potential regulator in the immunosuppressive microenvironment of hepatocellular carcinoma
Source: Genes Dis. 2024 Aug 28;12(3):101399. doi: 10.1016/j.gendis.2024.101399 (PMC11794160; doi:10.1016/j.gendis.2024.101399)
Supplement: Multimedia component 1 [file mmc1.docx]

**Supplementary methods**

***Data acquisition and processing***

***RNA-seq cohorts***

Multi-omics data including RNA-seq raw counts data, somatic mutation data, copy number variants (CNV) data, and clinical data, were retrieved from The Cancer Genome Atlas (TCGA) (https://tcga-data.nci.nih.gov/tcga/). Gene expression data and corresponding clinical data from the JP project were obtained from the International Cancer Genome Consortium (ICGC) (<https://dcc.icgc.org/>). Transcriptomics count data were converted to transcripts per kilobase million (TPM) values for further investigation.

***Microarray cohorts***

GSE14520 (n =488), and GSE54236 (n =81) with clinical prognostic information were achieved from Gene Expression Omnibus (GEO) (<https://www.ncbi.nlm.nih.gov/geo/>), as validation sets. For the GSE14520 and GSE54236, the raw data were normalized using R-package Affy's robust multi-array average (RMA) method and limma’s expression intensities normalization method, respectively. Microarray data of GEO cohorts was standardized across arrays, before being log2-transformed.

***Treatment cohorts***

GSE104580 (147 patients treated with TACE), GSE109211 (67 patients received sorafenib), and two immunotherapy cohorts; IMvigor210 cohort (n =298): metastatic urothelial carcinoma patients received anti-PD-1 therapy(1); Nathanson cohort (n =24): melanoma patients received anti-CTLA-4 therapy(2)). Therapeutic benefit information was generated from the series matrix files. The details of retrieved cohorts are displayed in **Table S1.**

**Lactate and immune-related genes**

A total of 284 lactate-related genes (LRGs) and 2013 immune-related genes (IRGs) were collected from the Molecular Signatures Database v7.5.1 (MSigDB, https://www.gsea-msigdb.org/) and the ImmPort database (https://www.immport.org/), respectively (**Table S2**). The TCGA-LIHC cohort was utilized to screen for differentially expressed genes (DEGs) between normal and tumor groups using a limma package with the thresholds of adjusted *P* <0.05 and |log2 fold change (log2FC)| >1. The ggvenn package was applied to identify overlapping genes of DEGs with LRGs and IRGs, respectively. Finally, the univariate Cox regression analysis was performed to detect prognostic-associated LRGs and IRGs for subsequent analysis.

**Identification and validation of lactate-immune-based subtypes (LIBS)**

Based on the iClusterplus package, we performed an integrative clustering evaluation for LRGs and IRGs to identify LIBS. The ideal quantity of clusters was decided primarily based on Bayesian information criteria (BIC). Additionally, clustering prediction index (CPI)(3) evaluation and assessment of the Gaps-statistics further identify the optimal number of clusters. Based on subtype-signature genes identified from linear models in the limma package, Nearest Template Prediction (NTP)(4) and subclass mapping analysis (SubMap)(5) were performed for validation in the GSE14520, GSE54236, and ICGC- LIRI-JP datasets.

***Associations of LIBS with clinical characteristics and preceding classifications***

We investigated the association of LIBS with clinical features, and the *ComplexHeatmap* package was utilized to visualize. Furthermore, univariate /multivariate Cox regression analyses were performed to determine independent prognostic factors. Three classical TCGA-LIHC classifications including iCluster subtype(6), Serum Tumor Biomarkers subtype(7), and Immunotype(8) were retrieved. The Sankey diagram was utilized to display the relationship between LIBS and previous classifications.

**Molecular characterization of LIBS**

To explore specific biological characteristics of LIBS, we conducted Gene Ontology (GO) and Kyoto Encyclopedia of Genes and Genomes (KEGG) analysis. Furthermore, a Gene set enrichment analysis (GSEA) algorithm was performed to explore the relationship between LIBS and cancer hallmark pathways. Gene sets were ordered by the normalized enrichment score (NES). By utilizing the single-sample genomic enrichment analysis (ssGSEA) algorithm, we investigated cancer-associated biological processes.

***Tumor microenvironment profiles***

Multiple algorithms encompassing CIBERSORT(9), Microenvironment Cell Populations-counter (MCP-counter)(10), xCell(11), Tumor Immune Estimation Resource (TIMER)(12), and Quantification of the Tumor Immune contexture from RNA-seq data (quanTIseq)(13) were utilized to calculate the level of immune and stromal cell subpopulation infiltration. Subsequently, immunogenicity was assessed by mapping immunograms according to previously published literature(14). Additionally, the expression patterns of 27 immune checkpoint molecules among the LIBS were further explored(15). SubMap analysis was applied to calculate the expression similarity between patients in the subgroups and patients who responded/non-responded to immune checkpoint inhibitors, thereby speculating immunotherapy efficacy(5). Furthermore, we applied several published algorithms containing (antigen presentation (APS)(16), and tumor inflammation signature (TIS)(17)) to quantify the efficacy of different subtypes of immunotherapy.TACE and sorafenib treatment cohorts were collected to evaluate the clinical efficacy among different subtypes.

***Genomic alterations***

To investigate genomic variation in the LIBS, we comprehensively examined mutation and CNV data from the TCGA-LIHC dataset. Afterward, we explored the differences among LIBS in the top 20 mutations and top 10 amplification (AMP) and homozygous deletion (HOMDEL) chromosomal regions via the maftools package. Further, we quantified phenotypic genomic alterations from the fraction of genome altered (FGA), fraction of genome gain (FGG), and fraction of genome lost (FGL) perspective(18, 19).

**Identification of the prognostic key gene**

To elucidate the underlying prognostic mechanisms of the dismal prognostic subtype, screening strategies for crucial biomarkers were considered from the perspective of signature genes. A two-step screening process was performed. Firstly, univariate Cox regression analysis with a threshold of HR >1, *P* <0.05 was utilized for the poorest subtype characteristic genes of enrolled cohorts, and the intersection of genes was used for subsequent analysis. Then, the area under the receiver operating characteristic curve (AUC) was calculated by the pROC package to identify the featured genes with the most predictive capability for the poorest prognostic subtype. The value with the highest average ROC statistic was deemed to be the diagnostic gene for LIBS1.

**Cell culture**

In this study, HCC cell lines (97H) treated with Dulbecco’s Modified Eagle’s medium (DMEM)/High-Glucose (Solarbio, Beijing, China) with 10% fetal bovine serum (Cyagen, Guangzhou, China) were maintained in a saturated humid incubator supplied with 5% CO2 at 37°C.

**Cell transfection and CEP55 silencing**

Small interfering RNA (siRNA) targeting CEP55 was utilized to generate an efficient knockdown. siRNA and negative control (NC) were synthesized by RiboBio (RiboBio, Guangzhou, China). Further, transfection efficiency was demonstrated by quantitative real-time polymerase chain reaction (qRT-PCR).

**Cell counting kit-8 (CCK-8) assay**

Transfected 97H cells were seeded in 96-well plates and cultured under suitable conditions for 24 h. Once the cells were adherent, the CCK-8 (US Everbright, Suzhou, China) assay was then conducted following the manufacturer’s instructions, and the absorbance at a wavelength of 450 nm was read using a microplate reader (Infinite F50, Tecan, Switzerland).

**5-Ethynyl-2'-deoxyuridine (EdU) incorporation assay**

Cell proliferation capacity was detected by 5-ethynyl-2′-deoxyuridine (EdU) incorporation assay. Pretreated 97H cells were processed with 50 μM EdU for 2 h at 37 °C. Next, the cells were fixed with 4% formaldehyde for 30 min and permeabilized with 0.5% Triton X-100 for 10 min. Further, each well was added 1×Apollo solution for 30 min and followed by 30 min of nuclei staining with 1×Hoechest 33342. Finally, the EdU-positive cells (red fluorescence) and Hoechst-positive cells (blue fluorescence) were visualized and calculated with a fluorescence microscope.

**Colony formation assay**

The transfected 97H cells were counted and seeded in 6-well plates (200 cells/well). The cells were in standardized culture for 7 to 10 days. Subsequently, colonies fixed with paraformaldehyde for 30 min were washed with phosphate-buffered saline (PBS) and stained with 0.1% crystal violet solution (Solarbio, Beijing, China) for 30 min. Finally, calculate the colony numbers after treatment for statistical analysis.

**Transwell migration assay**

Transwell plates (24-m pores) were used for Transwell migration. Next, 2.5 × 104 cells resuspended in serum-free medium were placed in the top chamber, and the lower compartment of complete medium with 10% FBS dulbecco's modified eagle medium (DMEM). The cells were fixed and stained after being incubated for 48 h at 37 °C. The migrated cells were counted with a light microscope.

**Scratch assay**

To assess the cell migration properties of *CEP55*, the scratch assay was conducted in 6-well plates. A straight line was scratched at the bottom of the well with a 200 uL pipette tip. Images at each edge of the gap were taken at time points 0 h and 24 h, respectively. The distance was measured to confirm the migration level.

**Statistical analysis**

R software version 4.1.3 was utilized for all data processing, statistical analysis, and visualization. Analysis of categorical variables was conducted by Pearson’s chi-squared test or Fisher’s exact test. The Kruskal-Wallis test compared continuous variables in more than two groups. Differences in survival among the LIBS were assessed by the Kaplan-Meier method and the log-rank test. The survival R package was utilized to perform Cox regression analysis. A two-tailed *P* value <0.05 was considered statistically significant.

**Reference**

1. Mariathasan S, Turley SJ, Nickles D, Castiglioni A, Yuen K, Wang Y, et al. TGFβ attenuates tumour response to PD-L1 blockade by contributing to exclusion of T cells. Nature. 2018;554(7693):544-8.

2. Nathanson T, Ahuja A, Rubinsteyn A, Aksoy BA, Hellmann MD, Miao D, et al. Somatic Mutations and Neoepitope Homology in Melanomas Treated with CTLA-4 Blockade. Cancer immunology research. 2017;5(1):84-91.

3. Chalise P, Fridley BL. Integrative clustering of multi-level 'omic data based on non-negative matrix factorization algorithm. PloS one. 2017;12(5):e0176278.

4. Hoshida Y. Nearest template prediction: a single-sample-based flexible class prediction with confidence assessment. PloS one. 2010;5(11):e15543.

5. Hoshida Y, Brunet JP, Tamayo P, Golub TR, Mesirov JP. Subclass mapping: identifying common subtypes in independent disease data sets. PloS one. 2007;2(11):e1195.

6. Comprehensive and Integrative Genomic Characterization of Hepatocellular Carcinoma. Cell. 2017;169(7):1327-41.e23.

7. Ahn KS, O'Brien DR, Kim YH, Kim TS, Yamada H, Park JW, et al. Associations of Serum Tumor Biomarkers with Integrated Genomic and Clinical Characteristics of Hepatocellular Carcinoma. Liver cancer. 2021;10(6):593-605.

8. Thorsson V, Gibbs DL, Brown SD, Wolf D, Bortone DS, Ou Yang TH, et al. The Immune Landscape of Cancer. Immunity. 2018;48(4):812-30.e14.

9. Newman AM, Liu CL, Green MR, Gentles AJ, Feng W, Xu Y, et al. Robust enumeration of cell subsets from tissue expression profiles. Nature methods. 2015;12(5):453-7.

10. Becht E, Giraldo NA, Lacroix L, Buttard B, Elarouci N, Petitprez F, et al. Estimating the population abundance of tissue-infiltrating immune and stromal cell populations using gene expression. Genome biology. 2016;17(1):218.

11. Aran D, Hu Z, Butte AJ. xCell: digitally portraying the tissue cellular heterogeneity landscape. Genome biology. 2017;18(1):220.

12. Li T, Fan J, Wang B, Traugh N, Chen Q, Liu JS, et al. TIMER: A Web Server for Comprehensive Analysis of Tumor-Infiltrating Immune Cells. Cancer research. 2017;77(21):e108-e10.

13. Finotello F, Mayer C, Plattner C, Laschober G, Rieder D, Hackl H, et al. Molecular and pharmacological modulators of the tumor immune contexture revealed by deconvolution of RNA-seq data. Genome medicine. 2019;11(1):34.

14. Kobayashi Y, Kushihara Y, Saito N, Yamaguchi S, Kakimi K. A novel scoring method based on RNA-Seq immunograms describing individual cancer-immunity interactions. Cancer science. 2020;111(11):4031-40.

15. Liu Z, Guo C, Li J, Xu H, Lu T, Wang L, et al. Somatic mutations in homologous recombination pathway predict favourable prognosis after immunotherapy across multiple cancer types. Clinical and translational medicine. 2021;11(12):e619.

16. Wang S, He Z, Wang X, Li H, Liu XS. Antigen presentation and tumor immunogenicity in cancer immunotherapy response prediction. eLife. 2019;8.

17. Damotte D, Warren S, Arrondeau J, Boudou-Rouquette P, Mansuet-Lupo A, Biton J, et al. The tumor inflammation signature (TIS) is associated with anti-PD-1 treatment benefit in the CERTIM pan-cancer cohort. Journal of translational medicine. 2019;17(1):357.

18. Liu Z, Liu L, Jiao D, Guo C, Wang L, Li Z, et al. Association of RYR2 Mutation With Tumor Mutation Burden, Prognosis, and Antitumor Immunity in Patients With Esophageal Adenocarcinoma. Frontiers in genetics. 2021;12:669694.

19. Liu Z, Xu H, Weng S, Ren Y, Han X. Stemness Refines the Classification of Colorectal Cancer With Stratified Prognosis, Multi-Omics Landscape, Potential Mechanisms, and Treatment Options. Frontiers in immunology. 2022;13:828330.
